# Autofusion in growing rod surgery for early onset scoliosis; what do we know so far?

**DOI:** 10.1051/sicotj/2024011

**Published:** 2024-04-30

**Authors:** Amr Hatem, Essam Mohamed Elmorshidy, Amer Elkot, Khaled Mohamed Hassan, Mohammad El-Sharkawi

**Affiliations:** 1 Orthopedics and Trauma Surgery, Faculty of Medicine, Assiut University Assiut Egypt; 2 Orthopedics and Trauma Surgery, Faculty of Medicine, Al-Azhar University Assiut Egypt

**Keywords:** Early onset scoliosis, Growing rods, Complications, Autofusion, Unplanned surgeries

## Abstract

The evolving landscape of *early onset scoliosis* management has shifted from the traditional paradigm of early definitive spinal fusion towards modern growth-friendly implants, particularly Growing Rods (GR). Despite the initial classification of GR treatment as a fusionless procedure, the phenomenon of autofusion has emerged as a critical consideration in understanding its outcomes. Studies have demonstrated the presence of autofusion since the early 1980s. The consequences of autofusion are extensive, impacting curve correction, diminishing trunk growth rate, and contributing to the “law of diminishing returns” in growing rod surgery. The literature suggests that autofusion may complicate definitive fusion surgery, leading to prolonged and intricate procedures involving multiple osteotomies. Additionally, it poses challenges in identifying anatomical landmarks during surgery, potentially increasing the risk of complications and revisions. While autofusion poses challenges to achieving optimal outcomes in growing rod treatment, it cannot be considered a standalone replacement for definitive fusion. Recent advances aim to limit autofusion and enhance treatment outcomes. In this review, we will delve into the existing literature on autofusion, examining studies that have documented its presence, probable causes, pathophysiology, potential implications for long-term patient outcomes, and possible new implants and techniques that decrease its incidence.

## Introduction

The management of progressive early-onset scoliosis (EOS) poses significant challenges, with its natural course marked by severe deformity, restrictive lung disease, and early mortality [1, 2]. Historically, the prevailing standard of care involved early definitive spinal fusion and instrumentation, guided by the belief that a short, straight spine was preferable to a longer, deformed one, despite the associated drawbacks of a short trunk. However, evolving principles in EOS treatment acknowledge that early thoracic spine fusion hampers spine and lung growth, leading to respiratory failure and heightened mortality [3]. Additionally, patients are dissatisfied with the cosmetic repercussions; specifically the disfigurement arising from disproportionate trunk-limb length [4, 5].

The surgical approach to progressive EOS has undergone substantial transformation through the adoption of growth-friendly implants. Growing Rods (GR), initially introduced by Moe and colleagues [6], aim to facilitate spinal and thoracic growth while managing curve progression to maintain optimal lung volume. Subsequent to their introduction, numerous modifications and advancements have been implemented globally, as evidenced by various studies [7, 8].

The conventional approach to growing-rod treatment involves the initial application, which accomplishes the majority of distraction and correction. Subsequent distractions are performed as frequently as every 6 months until skeletal maturity. Traditionally, the culmination of this process is definitive surgical fusion, with or without osteotomies. During this fusion, the growing rods are substituted with new instrumentation, often aiming for additional correction and fusion across the entire span, with no further plans for distraction or growth [9].

Contrary to its classification as a fusionless procedure, growing rods are linked to a high rate of autofusion, with some studies reporting rates as high as 89% [10–16]. The relevant studies reporting autofusion are shown in Table 1.

**Table 1:** Studies documenting the presence and implications of autofusion.

Study	Year	Authors	Design	Number of patients	Findings
Autofusion in the immature spine treated with growing rods [10].	2010	Cahill et al.	Retrospective, single centre	9	Rate of autofusion: 89% Average Cobb angle correction at the time of definitive fusion: 44% Average number of osteotomies per patient: 7
Avoidance of “final” surgical fusion after growing-rod treatment for early-onset scoliosis [15].	2016	Jain et al.	Retrospective, multicentre	167,137 underwent definitive fusion (final fusion group), 30 did not (observation group)	Average primary curve correction upon completion of treatment was 48% (from an initial average magnitude of 79° to a final average curve of 41°) No notable difference in the final curve magnitude between the two groups (41° in the observation group and 46° in the final surgical fusion group; *p* = 0.182)
Final fusion after growing-rod treatment for early onset scoliosis is it really final? [16].	2016	Poe-Kochert et al.	Retrospective, multicentre	100	30 complications necessitating reoperation (57 procedures) occurred in 20% of the patients On average, each patient experienced 1.5 complications following final fusion “Final” fusion may not actually be final
Graduation protocol after growing-rod treatment: removal of implants without new instrumentation is not a realistic approach [14].	2017	Kocyigit et al.	Prospective, single centre	26	90% of the patients in group 1 had progression of the deformity and this pathway was stopped due to ethical reasons
				Group 1 removed the rods without definitive fusion	
				Group 2 removed the rods and underwent definitive fusion	
Does the law of diminishing returns apply to the lengthening of the MCGR rod in early-onset scoliosis with reference to growth velocity? [17].	2017	Gardner et al.	Retrospective review of prospectively collected data, single-centre	28	No statistically significant difference in the amount of length achieved over the number of lengthening episodes

Remarkably, the current body of literature offers scant detailed elucidation of this particular phenomenon. Our objective with this article is to act as a catalyst, igniting further exploration into autofusion within the realm of growing rod surgery. Through our efforts to illuminate this phenomenon, we aspire to offer a wealth of knowledge that can significantly augment the treatment outcomes for children with early-onset scoliosis.

## Historical aspect

**Figure 1:**
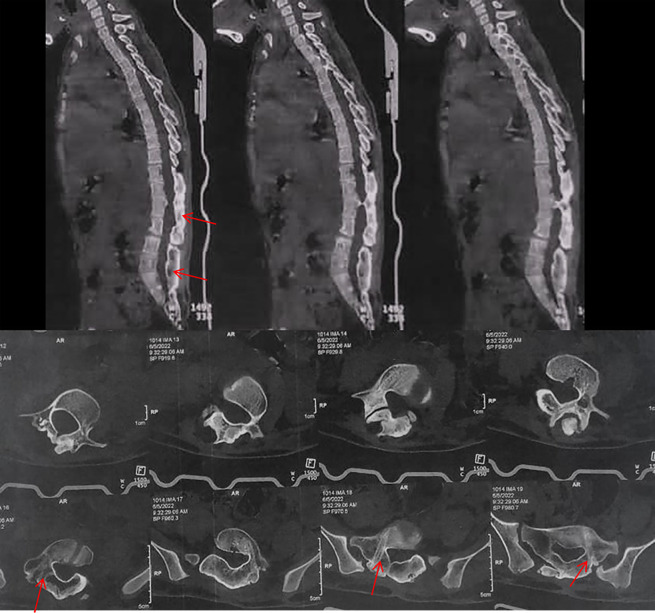
Axial and Sagittal CT cuts showing autofusion in a patient following growing rods removal, arrows pointing to the fusion mass and fused facets in the axial cuts.

The term “*autofusion*” was defined as the presence of a fusion mass at levels that were not intentionally fused before. This definition excluded the rostral and caudal ends of the construct, as fusion was anticipated or intended at these levels. The regions of autofusion exhibited a dense sheet of bone, resembling a mature fusion mass in a spine that had undergone previous intentional fusion (Figure 1) [10].

Autofusion in growth-friendly surgery has been reported in the literature since 1984 by Moe et al. [6] in children with subcutaneous *Harrington rod*, then in 1992 by Mardjetko et al. [18] when they noted spontaneous fusion in all 9 patients undergoing revision after Luque trolley instrumentation without fusion despite using an extraperiosteal exposure approach during the index procedure. The “spontaneous” fusion and the significant fibrosis presented obstacles to deformity correction, and addressing revisions proved to be a technically challenging task. Consequently, in situ reinstrumentation and arthrodesis were undertaken, resulting in preoperative and postoperative curves averaging 45° and 43°, respectively [18]. A mean blood loss of 1300 ml necessitated an average replacement of 2.5 units of blood. Moreover, these patients had already lost 75% of the initial correction after the first instrumentation making it a pretty upsetting scenario. Subsequently, Fisk et al. [13] reported 3 cases of spontaneous posterior fusion with subcutaneous insertion of the rods that led to the crankshaft phenomenon due to continued anterior growth and necessitated anterior fusion to control the deformity.

## Probable pathophysiology

The literature does not provide precise details regarding the exact pathophysiology of autofusion. It is postulated to be influenced by various factors, including immobilization, local disturbance of the perispinal musculature, periosteum, and soft tissues, direct contact between the rod and the spine, and the inherent ability of immature bone to quickly and reliably heal fractures, leading to the formation of spontaneous arthrodesis [10].

In a biochemical study by Huber et al., the *single-cell RNA* sequencing of the affected site by an injury (that may apply to soft tissue injury caused by EOS surgery) revealed an early upregulation of Mesenchymal Progenitor Cell genes linked to pathways involving cell adhesion and extracellular matrix-receptor interactions leading to the development of cartilage and bone [19]. Nevertheless, autofusion has also been documented in EOS surgeries not intimately related to the spine, such as expansion thoracoplasty [20, 21]. Betz et al. [22] demonstrated that there was no significant difference in the fusion rate between two groups of adolescent idiopathic scoliosis patients who underwent posterior spinal fusion with and without graft. This suggests that autofusion might be a typical physiological response to immobilization.

## Consequences

Comparable to early posterior spinal fusion, autofusion linked with growth-friendly surgeries may have the effect of constraining curve correction and diminishing trunk growth [23, 24]. Autofusion is posited to contribute to the phenomenon known as “*The law of diminishing returns*” in growing rod surgery, where repeated lengthenings are associated with a decrease in the length gained with each successive procedure [25]. In the study by Sankar et al., the Cobb angle showed a reduction from an initial mean of 74°–36° after the primary implantation, but did not significantly change with repeated lengthenings (*P* > 0.96). Additionally, the T1-S1 gain following each subsequent lengthening exhibited a significant decrease (*P* < 0.007). In a study conducted by Noordeen et al. [26], intraoperative measurement of distraction forces was performed in 60 consecutive lengthenings across 26 patients. The results revealed a significant increase in distraction force during the fifth lengthening (mean 368 N ± 54 N) compared to the preceding lengthening (*P* < 0.01). Moreover, the mean length achieved at each distraction decreased progressively over time, reaching a point where consistently 8 mm or less was attained by the fifth lengthening.

The effect of autofusion on trunk height is rather unclear in the literature. In spite of the law of diminishing returns, the mean T1-S1 gain during the growing rod treatment period is equated to the average T1-S1 gain in normal children [11, 25, 27]. This phenomenon could be elucidated by the biological activity of the fusion mass, which may respond to distractive stresses during lengthening [25]. Campbell et al. [28] observed an increase in the length of a unilateral unsegmented bar in response to VEPTR lengthening. On the other hand, in the *Luque trolley technique*, the increases in trunk height were noted to be less than what would be predicted as normal. The children gained only 35% of the anticipated growth over the spanned levels [18].

Cahill et al. [10] reported a 44% Cobb angle correction at the final fusion, while Akbarnia et al. [11] found corrections of only 24% at the final definitive fusion in children with growing rods. This is in contrast to a 71.2% correction [29], 79.1% [30], 73.7% [31], and 56.8% in severe idiopathic scoliosis (Cobb angle > 70°–90°) [32] in children who did not undergo previous instrumentation. In a systematic review by Ahuja et al. [33], the degree of curve correction achieved during the definitive fusion procedure was described as “modest”, primarily attributed to autofusion and the spinal rigidity developed throughout the distraction treatment.

Correction, albeit modest, is attained through rigorous and prolonged procedures involving multiple osteotomies. In a study by Vittoria et al. [34], out of 40 patients undergoing definitive fusion after growing rods, 15 patients required at least one osteotomy. Similarly, Cahill et al. [10] reported performing an average of 7 Smith-Petersen osteotomies to achieve a Cobb angle correction of 44% at the time of definitive surgery. In the paper by Flynn et al. [24], osteotomies were performed in 22 (24%) of the patients who underwent a final procedure. Additionally, anterior fusion or anterior release was reported for 12 (13%) patients.

Furthermore, the presence of autofusion complicates the identification of anatomical bony landmarks, rendering the surgical process more intricate. This complexity may extend the duration of definitive fusion surgery, and potentially raise the theoretical risk of screws misplacement, neurological complications, and the need for revisions. In a multicenter study including 167 patients with traditional growing rods for risk factors for reoperation following final fusion by Du et al. [35], univariate analysis revealed that patients necessitating revision surgery after final fusion had a lengthier treatment duration with traditional growing rods. Furthermore, it was found in the multivariate analysis that the number of levels spanned with traditional growing rods and the duration of treatment with traditional growing rods was independently associated with the need for revision surgery after final fusion.

There is sparse data on the frequency of autofusion in patients with *magnetically controlled growing rods* (*MCGRs*). MCGRs are proposed to be less associated with autofusion for two basic reasons: a decreased number of surgeries and thus less trauma to the posterior spinal elements and musculature, and more frequent lengthenings of smaller magnitude maintaining a more sustained, long-term distractive force [36]. Gardner et al. demonstrated that the “law of diminishing returns” does not impact the serial lengthenings of MCGRs in the manner observed with traditional growing rods. There was no statistically significant difference in the length gain achieved over the number of lengthening episodes (*P* = 0.427) for a period of at least 2 years [37]. Nevertheless, incidents of autofusion with MCGRs have been reported to the best of our knowledge in at least three studies. The first instance involved a patient with *Ehlers-Danlos Syndrome* who had multiple reoperations. The second case was observed in a patient with *Prader-Willi syndrome*, and the third case involved a patient with tetraplegic cerebral palsy [36, 38, 39]. The three studies thus have a predisposing factor for autofusion.

The significant rate of complications observed in final fusion surgery after growing rod treatment, possibly influenced by autofusion, has prompted a reconsideration of the term “final” in the context of definitive fusion surgery. In a retrospective study, Poe-Kochert et al. [16] reviewed 100 patients over a 2-year follow-up period post the supposed “final fusion” procedure. Out of the study cohort, 20% experienced 30 complications, averaging 1.5 complications per patient, necessitating a total of 57 reoperations. The average time to the first reoperation following the “final fusion” was 2.0 years. The researchers concluded that caution is warranted when advising parents, highlighting that the term “final fusion” may not accurately convey the last surgical intervention needed for the comprehensive and permanent correction of spinal deformities.

## Can autofusion offer a standalone replacement for definitive fusion?

Autofusion, while hindering correction at the time of definitive fusion, cannot be regarded as a substitute for definitive fusion in all cases. Kocyigit et al. [14] studied 26 patients identified at the age of 14 with comprehensive medical records, regular and uncomplicated lengthening procedures, and a minimum 2-year follow-up. They divided the patients into 2 groups; the 1st with stable radiographs underwent removal of the growing rods without instrumented fusion. The second group lacking sufficient correction underwent the removal of the growing rods along with instrumented fusion. Out of the ten patients in group 1, nine exhibited notable deformity progression following the extraction of growing rods, while one remained stable. The treatment pathway for group 1 was prematurely halted for ethical reasons due to its elevated failure rate. The authors firmly concluded that the removal of growing rods with no further procedures leads to an unacceptably high rate of deformity progression affirming that an extended course of growing-rod treatment does not invariably lead to automatic and dependable fusion. Additionally, Jain et al. [40] observed an escalation in the curvature magnitude among patients requiring implant removal due to infection. They explained this by proposing several theories. Firstly, autofusion may not be complete in all segments, with some retaining motion. Secondly, the fusion mass may be thin, a common finding during definitive fusion. This thin fusion mass might lack durability, making it susceptible to breaking or stretching under stress [40]. As no significant difference was found between patients undergoing final fusion and those retaining the implant with regard to the final curve magnitude, the authors concluded that maintaining implants serves as an acceptable endpoint for growing-rod treatment in patients with acceptable final alignment and trunk height, minimal length gain at the last distraction, and no apparent clinical or radiographic issues related to the implant, while fusion becomes imperative in patients exhibiting unsatisfactory sagittal or coronal parameters, in patients experiencing curve progression during GR treatment, and if the implants are removed due to infection or other complications [40].

## Possible advances to limit autofusion

In an attempt to address autofusion in GRs, two recently developed growing rod systems have been introduced and studied. These include the *Semiconstrained Growing Rods* (*SCGRs*) *and the minimally invasive bipolar technique.* The studies examining these two systems are shown in Table 2.

**Table 2:** Studies examining modern advances in growing rods.

Study	Year	Authors	Design	Number of patients	Findings
Minimizing spine autofusion with the use of semiconstrained growing rods for early onset scoliosis in children [41].	2018	Bouthors et al.	Prospective, single centre	28, 18 underwent definitive fusion.	The change in the angle of the major curve and trunk length (T1-S1) solely due to the final fusion surgery (which reflects the spine’s mobility at the time of fusion surgery) averaged 20.3° (SD, 16.1; range 5–60) and 31.7 mm (SD, 23.1; range, 10–96), respectively
Minimally invasive surgery for neuromuscular scoliosis [42].	2018	Miladi et al.	Retrospective, single centre	100 patients – bipolar construct anchored proximally by hooks in a double claw and distally by iliosacral screws through a minimally invasive approach.	Cobb angle improved from 89.8° to 35.8° which corresponds to 61% correction at the latest follow up(average 3 years, range 2–6 years) Mean preoperative hyper kyphosis was reduced from 68° to 33° 26 complications

The SCGRs are a new generation of growing rods offering the added benefit of axial rotation freedom within its components. They are proposed to diminish autofusion in vivo, consequently optimizing coronal plane correction, T1-S1 growth, and the ultimate correction attained during definitive fusion for children with early-onset scoliosis. In the study by Bouthors et al. [41], the patients demonstrated a statistically significant increase in T1-S1 trunk length and a statistically significant decrease in the severity of scoliosis throughout the course of GR treatment. The definitive surgery alone resulted, without the need to perform osteotomies, in a correction of the major Cobb curve angle by a mean of 20.3° (36.8%) and an increase in the T1-S1 trunk length of a mean of 31.7 mm. This suggests that autofusion had been minimized, with relatively low complication rates, as evidenced by 14 complications involving 11 of the 28 patients giving a mean rate of 0.096 complications per patient per year. This rate of complications is lower than what has been reported in the literature for traditional GRs or MCGRs, which was 0.15 complications per patient per year for traditional GRs and 0.32 complications per patient per year for MCGRs in a series by Teoh et al. [43] and even higher at a rate of 2.06 complications per patient by Sanker et al. [44]. The Minimally Invasive Bipolar Technique relies on the gradual internal correction of the deformity through the viscoelastic relaxation of the trunk. This bipolar method involves constructing a telescopic structure that spans the curve and maintains constant tension between the two ends. Proximal fixation is achieved using two supra-laminar pedicle hook claws on each side, spanning four or five adjacent vertebrae. Distal fixation is accomplished using pedicle screws in two or three levels on each side for idiopathic or syndromic scoliosis, or iliosacral screws in the cases of neuromuscular scoliosis [45]. The anchors at both ends of the construct need to be connected by a robust link, typically one or two rods with a diameter of 5.5 mm or more. This connection is established through a minimally invasive approach, involving two small incisions located in front of the spine’s fixation zones. This method is designed to circumvent work in the intermediate zone, effectively minimizing the risk of fibrosis and spontaneous fusion. This approach has demonstrated favourable clinical and radiological outcomes with minimal associated morbidity in the management of neuromuscular spine deformities [46]. However, additional research is warranted to further explore its efficacy and potential benefits.

## Authors commentary

In our centre, we operated a fair number of children with EOS using growing rods starting in 2009, with favourable short-term results [47, 48]. Among these, 11 patients underwent definitive fusion. Notably, in these cases, definitive fusion surgeries unveiled a significant occurrence of autofusion, which introduced complexities into the surgical procedures and resulted in various challenges and complications, including severe issues such as screw misplacement and postoperative paraplegia.

The primary aim of this article is to thoroughly investigate and comprehensively understand the potential underlying factors contributing to autofusion and to stimulate additional investigation into the concept of autofusion within the context of growing rod surgery in order to inspire researchers to develop innovative tools, techniques and methods aimed at reducing and mitigating its occurrence. By shedding light on this phenomenon, we intend to provide valuable insights that have the potential to greatly enhance the treatment effectiveness for children diagnosed with early-onset scoliosis.

## Conclusion

Exploration of autofusion in growing rod surgery reveals its multifaceted impact on treatment outcomes for early-onset scoliosis. The historical perspective underscores the longstanding recognition of autofusion, while its probable pathophysiology points to various factors influencing unintended fusion. The consequences of autofusion extend beyond curve correction challenges to complications in definitive fusion surgery, prompting a critical reassessment of the term “final fusion”. Despite hindering correction and possibly growth of the trunk, and contributing to “the law of diminishing returns” autofusion cannot serve as a standalone alternative to definitive fusion. Innovative approaches like Semiconstrained Growing Rods and the Minimally Invasive Bipolar Technique offer promising avenues for minimizing autofusion and optimizing patient outcomes. Continued research and technological evolution are vital for refining strategies in the dynamic landscape of early-onset scoliosis management.

## Data Availability

This is a review of the literature, no patient data were collected. the only patient data used were the ct images in Figure 1, parental consent was obtained for using the patients’ radiological images in scientific publications.

## References

[1] Pehrsson K, Larsson S, Oden A, Nachemson A (1992) Long-term follow-up of patients with untreated scoliosis. A study of mortality, causes of death, and symptoms. Spine (Phila Pa 1976) 17(9), 1091–1096. 1411763 10.1097/00007632-199209000-00014

[2] Fernandes P, Weinstein SL (2007) Natural history of early onset scoliosis. J Bone Joint Surg Am 89(Suppl 1), 21–33. 17272420 10.2106/JBJS.F.00754

[3] Karol LA, Johnston C, Mladenov K, Schochet P, Walters P, Browne RH (2008) Pulmonary function following early thoracic fusion in non-neuromuscular scoliosis. JBJS 90(6), 1272–1281. 10.2106/JBJS.G.00184 18519321

[4] Goldberg CJ, Gillic I, Connaughton O, Moore DP, Fogarty EE, Canny GJ, Dowling FE (2003) Respiratory function and cosmesis at maturity in infantile-onset scoliosis. Spine (Phila Pa 1976) 28(20), 2397–2406. 14560091 10.1097/01.BRS.0000085367.24266.CA

[5] Winter RB, Moe JH (1982) The results of spinal arthrodesis for congenital spinal deformity in patients younger than five years old. JBJS 64(3), 419–432. 7061559

[6] Moe JH, Kharrat K, Winter RB, Cummine JL (1984) Harrington instrumentation without fusion plus external orthotic support for the treatment of difficult curvature problems in young children. Clin Orthop Relat Res 185, 35–45. 6705397

[7] Thompson GH, Akbarnia BA, Kostial P, Poe-Kochert C, Armstrong DG, Roh J, et al. (2005) Comparison of single and dual growing rod techniques followed through definitive surgery: A preliminary study. Spine (Phila Pa 1976) 30(18), 2039–2044. 16166892 10.1097/01.brs.0000179082.92712.89

[8] Klemme WR, Denis F, Winter RB, Lonstein JW, Koop SE (1997) Spinal instrumentation without fusion for progressive scoliosis in young children. J Pediatr Orthop 17(6), 734–742. 9591974

[9] Akbarnia BA, Marks DS, Boachie-Adjei O, Thompson AG, Asher MA (2005) Dual growing rod technique for the treatment of progressive early-onset scoliosis: A multicenter study. Spine 30(17S), S46–S57. 16138066 10.1097/01.brs.0000175190.08134.73

[10] Cahill PJ, Marvil S, Cuddihy L, Schutt C, Idema J, Clements DH, et al. (2010) Autofusion in the immature spine treated with growing rods. Spine (Phila Pa 1976) 35(22), E1199–E1203. 20683383 10.1097/BRS.0b013e3181e21b50

[11] Akbarnia BA, Breakwell LM, Marks DS, McCarthy RE, Thompson AG, Canale SK, et al. (2008) Dual growing rod technique followed for three to eleven years until final fusion: The effect of frequency of lengthening. Spine (Phila Pa 1976) 33(9), 984–990. 18427320 10.1097/BRS.0b013e31816c8b4e

[12] Akbarnia BA, Marks DS, Boachie-Adjei O, Thompson AG, Asher MA (2005) Dual growing rod technique for the treatment of progressive early-onset scoliosis: A multicenter study. Spine (Phila Pa 1976) 30(17 Suppl), S46–S57. 16138066 10.1097/01.brs.0000175190.08134.73

[13] Fisk JR, Peterson HA, Laughlin R, Lutz R (1995) Spontaneous fusion in scoliosis after instrumentation without arthrodesis. J Pediatr Orthop 15(2), 182–186. 7745090

[14] Kocyigit IA, Olgun ZD, Demirkiran HG, Ayvaz M, Yazici M (2017) Graduation protocol after growing-rod treatment: Removal of implants without new instrumentation is not a realistic approach. J Bone Joint Surg Am 99(18), 1554–1564. 28926385 10.2106/JBJS.17.00031

[15] Jain A, Sponseller PD, Flynn JM, Shah SA, Thompson GH, Emans JB, et al. (2016) Avoidance of “final” surgical fusion after growing-rod treatment for early-onset scoliosis. J Bone Joint Surg Am 98(13), 1073–1078. 27385680 10.2106/JBJS.15.01241

[16] Poe-Kochert C, Shannon C, Pawelek JB, Thompson GH, Hardesty CK, Marks DS, et al. (2016) Final fusion after growing-rod treatment for early onset scoliosis: Is it really final? J Bone Joint Surg Am 98(22), 1913–1917. 27852908 10.2106/JBJS.15.01334

[17] Gardner A, Beaven A, Marks D, Spilsbury J, Mehta J, Newton Ede M (2017) Does the law of diminishing returns apply to the lengthening of the MCGR rod in early onset scoliosis with reference to growth velocity? J Spine Surg 3(4), 525–530. 29354727 10.21037/jss.2017.08.16 PMC5760426

[18] Mardjetko SM, Hammerberg KW, Lubicky JP, Fister JS (1992) The Luque trolley revisited. Review of nine cases requiring revision. Spine (Phila Pa 1976) 17(5), 582–589. 1621159 10.1097/00007632-199205000-00018

[19] Huber AK, Patel N, Pagani CA, Marini S, Padmanabhan KR, Matera DL, et al. (2020) Immobilization after injury alters extracellular matrix and stem cell fate. J Clin Invest 130(10), 5444–5460. 32673290 10.1172/JCI136142 PMC7524473

[20] Yilgor C, Demirkiran G, Ayvaz M, Yazici M (2012) Is expansion thoracoplasty a safe procedure for mobility and growth potential of the spine? Spontaneous fusion after multiple chest distractions in young children, J Pediatr Orthop 32(5), 483–489. 22706464 10.1097/BPO.0b013e318257d3a9

[21] Lattig F, Taurman R, Hell AK (2016) Treatment of early-onset spinal deformity (EOSD) with VEPTR: A challenge for the final correction spondylodesis – a case series clinical spine. Surgery 29(5), E246–E251. 10.1097/BSD.0b013e31826eaf27 27196004

[22] Betz RR, Petrizzo AM, Kerner PJ, Falatyn SP, Clements DH, Huss GK (2006) Allograft versus no graft with a posterior multisegmented hook system for the treatment of idiopathic scoliosis. Spine (Phila Pa 1976) 31(2), 121–127. 16418628 10.1097/01.brs.0000194771.49774.77

[23] Sawyer JR, de Mendonça RG, Flynn TS, Samdani AF, El-Hawary R, Spurway AJ, et al. (2016) Complications and radiographic outcomes of posterior spinal fusion and observation in patients who have undergone distraction-based treatment for early onset scoliosis. Spine Deform 4(6), 407–412. 27927569 10.1016/j.jspd.2016.08.007

[24] Flynn JM, Tomlinson LA, Pawelek J, Thompson GH, McCarthy R, Akbarnia BA (2013) Growing-rod graduates: Lessons learned from ninety-nine patients who completed lengthening. J Bone Joint Surg Am 95(19), 1745–1750. 24088966 10.2106/JBJS.L.01386

[25] Sankar WN, Skaggs DL, Yazici M, Johnston CE 2nd, Shah SA, Javidan P, et al. (2011) Lengthening of dual growing rods and the law of diminishing returns. Spine (Phila Pa 1976) 36(10), 806–809. 21336236 10.1097/BRS.0b013e318214d78f

[26] Noordeen HM, Shah SA, Elsebaie HB, Garrido E, Farooq N, Al Mukhtar M (2011) In vivo distraction force and length measurements of growing rods: Which factors influence the ability to lengthen? Spine 36(26), 2299–2303. 21494191 10.1097/BRS.0b013e31821b8e16

[27] Cheung KM, Cheung JP, Samartzis D, Mak KC, Wong YW, Cheung WY, et al. (2012) Magnetically controlled growing rods for severe spinal curvature in young children: A prospective case series. Lancet 379(9830), 1967–1974. 22520264 10.1016/S0140-6736(12)60112-3

[28] Campbell RM, Hell-Vocke AK (2003) Growth of the thoracic spine in congenital scoliosis after expansion thoracoplasty. J Bone Joint Surg Am 85, 409–420. 12637424 10.2106/00004623-200303000-00002

[29] Storer SK, Vitale MG, Hyman JE, Lee FY, Choe JC, Roye DP Jr (2005) Correction of adolescent idiopathic scoliosis using thoracic pedicle screw fixation versus hook constructs. J Pediatr Orthop 25(4), 415–419. 15958886 10.1097/01.mph.0000165134.38120.87

[30] Elnady B, El-Sharkawi MM, El-Meshtawy M, Adam FF, Said GZ (2017) Posterior-only surgical correction of adolescent idiopathic scoliosis: An Egyptian experience. SICOT-J 3, 69. 29227788 10.1051/sicotj/2017057 PMC5725150

[31] Elnady B, El-Sharkawi M, El-Meshtawy M, Adam F, Hassan K (2015) High density pedicle screws through posterior only approach for surgical correction of severe adolescent idiopathic scoliosis> 70o Egyptian. Spine J 15, 37–44.

[32] Mihara Y, Chung WH, Mohamad SM, Chiu CK, Chan CYW, Kwan MK (2021) Predictive factors for correction rate in severe idiopathic scoliosis (Cobb angle ≥ 90°): An analysis of 128 patients. Eur Spine J 30(3), 653–660. 33486626 10.1007/s00586-020-06701-3

[33] Ahuja K, Ifthekar S, Mittal S, Bali SK, Yadav G, Goyal N, et al. (2023) Is final fusion necessary for growing-rod graduates: A systematic review and meta-analysis. Global Spine J 13(1), 209–218. 35410498 10.1177/21925682221090926 PMC9837500

[34] Vittoria F, Ceconi V, Fantina L, Barbi E, Carbone M (2022) Effectiveness and safety of a one-yearly elongation approach of growing rods in the treatment of early-onset scoliosis: A case series of 40 patients with definitive fusion. Front Pediatr 10, 895065. 36467489 10.3389/fped.2022.895065 PMC9715965

[35] Du JY, Poe-Kochert C, Thompson GH, Hardesty CK, Pawelek JB, Flynn JM, Emans JB (2020) Risk factors for reoperation following final fusion after the treatment of early-onset scoliosis with traditional growing rods. J Bone Joint Surg Am 102(19), 1672–1678. 33027120 10.2106/JBJS.20.00312

[36] Yang MJ, Rompala A, Samuel SP, Samdani A, Pahys J, Hwang S (2023) Autofusion with magnetically controlled growing rods: A case report. Cureus 15(3), e36638. 37155436 10.7759/cureus.36638 PMC10122916

[37] Gardner A, Beaven A, Marks D, Spilsbury J, Mehta J, Ede MN (2017) Does the law of diminishing returns apply to the lengthening of the MCGR rod in early onset scoliosis with reference to growth velocity? J Spine Surg 3(4), 525–530. 29354727 10.21037/jss.2017.08.16 PMC5760426

[38] Cheung JPY, Sze KY, Cheung KMC, Zhang T (2021) The first magnetically controlled growing rod (MCGR) in the world – lessons learned and how the identified complications helped to develop the implant in the past decade: Case report. BMC Musculoskelet Disord 22(1), 319. 33794851 10.1186/s12891-021-04181-0 PMC8015050

[39] Green AH, Brzezinski A, Ishmael T, Adolfsen S, Bowe JA (2021) Premature spinal fusion after insertion of magnetically controlled growing rods for treatment of early-onset scoliosis: Illustrative case. J Neurosurg Case Lessons 2(17), CASE21446. 36060899 10.3171/CASE21446 PMC9435560

[40] Jain A, Sponseller PD, Flynn JM, Shah SA, Thompson GH, Emans JB, et al. (2016) Avoidance of “final” surgical fusion after growing-rod treatment for early-onset scoliosis. J Bone Joint Surg Am 98(13), 1073–1078. 27385680 10.2106/JBJS.15.01241

[41] Bouthors C, Izatt MT, Adam CJ, Pearcy MJ, Labrom RD, Askin GN (2018) Minimizing spine autofusion with the use of semiconstrained growing rods for early onset scoliosis in children. J Pediatr Orthop 38(10), e562–e571. 30199457 10.1097/BPO.0000000000001242

[42] Miladi L, Gaume M, Khouri N, Johnson M, Topouchian V, Glorion C (2018) Minimally invasive surgery for neuromuscular scoliosis: Results and complications in a series of one hundred patients. Spine (Phila Pa 1976) 43(16), e968–e975. 29419720 10.1097/BRS.0000000000002588 PMC6080881

[43] Teoh KH, Winson DM, James SH, Jones A, Howes J, Davies PR, Ahuja S (2016) Do magnetic growing rods have lower complication rates compared with conventional growing rods? Spine J 16(4 Suppl), S40–S44. 26850175 10.1016/j.spinee.2015.12.099

[44] Sankar WN, Acevedo DC, Skaggs DL (2010) Comparison of complications among growing spinal implants. Spine (Phila Pa 1976) 35(23), 2091–2096. 20562733 10.1097/BRS.0b013e3181c6edd7

[45] Miladi L (2020) The minimally invasive bipolar technique for the treatment of spinal deformities in children and adolescents. Coluna/Columna 19, 308–313.

[46] Oliveira R, Defino H, Costa H (2021) Preliminary results of the bipolar technique in the treatment of neuromuscular scoliosis. Coluna/Columna 20, 169–173.

[47] El-Sharkawi M, Koptan W, Shawky A, Mostafa A, Tammam H, Gad W, Aboloyoun N (2016) Management of early onset scoliosis using growing spine profiler (GSP). Global Spine J 6, s-0036.

[48] El-Sharkawi MM, Alkot A (2021) Egyptian experience of surgical management of early-onset scoliosis. CRC Press.

